# Changes in Respiratory Muscle Strength Following Cardiac Rehabilitation for Prognosis in Patients with Heart Failure

**DOI:** 10.3390/jcm9040952

**Published:** 2020-03-30

**Authors:** Nobuaki Hamazaki, Kentaro Kamiya, Shohei Yamamoto, Kohei Nozaki, Takafumi Ichikawa, Ryota Matsuzawa, Shinya Tanaka, Takeshi Nakamura, Masashi Yamashita, Emi Maekawa, Kentaro Meguro, Chiharu Noda, Minako Yamaoka-Tojo, Atsuhiko Matsunaga, Junya Ako

**Affiliations:** 1 Department of Rehabilitation, Kitasato University Hospital, Sagamihara 252-0375, Japan; 2 Department of Rehabilitation, Kitasato University School of Allied Health Sciences, Sagamihara 252-0373, Japan; 3 Department of Rehabilitation Sciences, Kitasato University Graduate School of Medical Sciences, Sagamihara 252-0373, Japan; 4 Department of Epidemiology and Prevention, Center for Clinical Sciences, National Center for Global Health and Medicine, Tokyo 162-8655, Japan; 5 Department of Physiotherapy, School of Rehabilitation, Hyogo University of Health Sciences, Kobe 650-8530, Japan; 6 Department of Rehabilitation, Nagoya University Medical School Hospital, Nagoya 466-8560, Japan; 7 Department of Cardiovascular Medicine, Kitasato University School of Medicine, Sagamihara 252-0373, Japan

**Keywords:** change in respiratory muscle strength, heart failure, cardiac rehabilitation, prognosis, clinical event

## Abstract

Respiratory muscle weakness, frequently observed in patients with heart failure (HF), is reported as a predictor for poor prognosis. Although increased respiratory muscle strength ameliorates exercise tolerance and quality of life in HF patients, the relationship between changes in respiratory muscle strength and patient prognosis remains unclear. A total of 456 patients with HF who continued a 5-month cardiac rehabilitation (CR) were studied. We measured maximal inspiratory pressure (PI_max_) at hospital discharge as the baseline and five months thereafter to assess the respiratory muscle strength. Changes in PI_max_ during the 5-month observation period (⊿PI_max_) were examined. We investigated the composite multiple incidence of all-cause death or unplanned readmission after 5-month CR. The relationship between ⊿PI_max_ and the incidence of clinical events was analyzed. Over a median follow-up of 1.8 years, 221 deaths or readmissions occurred, and their rate of incidence was 4.3/100 person-years. The higher ⊿PI_max_ was significantly associated with lower incidence of clinical event. In multivariate Poisson regression model after adjustment for clinical confounding factors, ⊿PI_max_ remained a significant and independent predictor for all-cause death/readmission (adjusted incident rate ratio for ⊿PI_max_ increase of 10 cmH_2_O: 0.77, 95% confidence interval: 0.70–0.86). In conclusion, the changes in respiratory muscle strength independently predict the incidence of clinical events in patients with HF.

## 1. Introduction

Patients with heart failure (HF) frequently suffer from breathlessness during exercise, leading to exercise intolerance and decreased quality of life [1]. Exercise-related breathlessness partly results from respiratory muscle weakness, observed in approximately 30–50% of patients with HF [2]. The respiratory muscle weakness is caused by muscular atrophy and/or decreased number of cross-bridges, resulting from the activation of inflammatory and neuroendocrine factors due to HF [2,3,4,5]. Additionally, several studies have demonstrated that respiratory muscle weakness is an independent predictor of exercise intolerance, ventilatory inefficiency during exercise, and poor prognosis of patients with HF [6,7]. However, the influence of longitudinal changes in respiratory muscle strength on prognostic outcomes remains unclear.

Conversely, comprehensive cardiac rehabilitation including exercise training, medication administration, nutritional management, and correcting lifestyle behavior is recognized as one of the key treatment strategies to prevent HF recurrence and to improve prognosis of patients with HF [8]. Furthermore, respiratory muscle training is reported to increase respiratory muscle strength and consequently improve exercise tolerance and quality of life in HF patients [9]. Hence, the current guideline on preventive cardiology and rehabilitation has recommended that respiratory training should be prescribed to patients with HF with exercise intolerance and respiratory muscle weakness in addition to the usual exercise training [10]. However, the effect of increased respiratory muscle strength on prognosis is unclear, although decreased respiratory muscle strength is reportedly associated with HF severity [11]. Our hypothesis was that the changes in respiratory muscle strength might potentially be a useful marker to assess the clinical status of HF as well as a surrogate marker to predict outcomes in patients with HF.

Therefore, this study aimed to investigate the relationship between changes in respiratory muscle strength following cardiac rehabilitation and the incidence of adverse clinical events in patients with HF.

## 2. Materials and Methods

### 2.1. Study Design and Population

This is a single-center observational study conducted to review a cohort of consecutive patients with HF who were admitted to Kitasato University Hospital for HF treatment and underwent a 5-month cardiac rehabilitation during hospitalization and after hospital discharge from May 2009 to December 2017. Patients who had received thoracic or open-heart surgery within the last three months or had chronic diseases of respiratory systems were excluded from the study. Comprehensive cardiac rehabilitation consisted of supervised exercise training and education on self-management including medication, nutrition, and physical activity, based on the statement from the Japanese Circulation Society [12]. Blood examinations and echocardiograms at hospital discharge were considered as baseline data. We also assessed pulmonary and respiratory muscle functions at baseline and at the end of the 5-month cardiac rehabilitation. Events of all-cause mortality or all-cause unplanned readmission after the 5-month cardiac rehabilitation were considered as the primary end-point of this study. Data on all variables were obtained from an electronic database. The study protocol was approved by the Kitasato Institute Clinical Research Review Board (KMEO B18-075, September 4, 2018) and was performed according to the ethical guidelines of the Declaration of Helsinki.

### 2.2. Patient Characteristics

Data on age, gender, body mass index (BMI), HF severity assessed by the New York Heart Association functional classification (NYHA class), smoking history, medications, and medical history such as hypertension, diabetes mellitus, dyslipidemia, chronic kidney disease, or atrial fibrillation were obtained from medical records upon study participation. Routine laboratory analysis included hemoglobin and serum albumin as well as plasma brain natriuretic peptide (BNP). The estimated glomerular filtration rate (eGFR) was determined by serum creatinine levels. The left ventricular ejection fraction (LVEF) was also measured on echocardiograms using the 2D method. The AHEAD score was used to assess the patients’ risk stratification and was calculated by assigning one point to the patient for each of the following factors: A: atrial fibrillation, H: hemoglobin <13 g/dL for men and 12 g/dL for women, E: elderly (age >70 years), A: abnormal renal parameters (creatinine >130 µmol/dL), and D: diabetes mellitus [13]. The functional capacity was measured using the 6-min walk distance according to standard guidelines [14].

### 2.3. Pulmonary and Respiratory Muscle Functions

To assess the pulmonary function, spirometry without bronchodilator was performed to measure forced vital capacity (FVC) and forced expiratory volume in 1 s (FEV_1_) using a spirometer (Autospiro AS-507, Minato Medical Science, Osaka, Japan), and their percentages were calculated relative to the predictive values issued by the Japanese Respiratory Society [15]. To assess the respiratory muscle function, we measured the maximal inspiratory pressure (PI_max_) using a pressure transducer (Autospiro AAM-377, Minato Medical Science, Osaka, Japan) connected to the spirometer, according to the joint statement of the American Thoracic Society and European Respiratory Society [16]. In the measurement of PI_max_, patients in a sitting position were instructed to hold a 25-mm-diameter flanged mouthpiece in their mouth and perform a 3-s forced inspiration from the level of maximal expiration. PI_max_ was determined by the average value of the maximum pressure over a 1-s period during the 3-s forced inspiration. In this study, PI_max_ was expressed as its absolute value, although it showed negative pressure for atmospheric pressure. The measurements were performed three times, and the maximum value in PI_max_ was accepted for analysis. We also calculated percentage PI_max_ (% PI_max_) relative to the predictive value that was estimated using each age, gender, height, and body weight [17]. The % PI_max_ of <70% was defined as respiratory muscle weakness based on previous reports in patients with HF [2,7,18].

### 2.4. End-Points

The primary end-point of this study was a composite of multiple all-cause clinical events including all-cause death and/or all-cause unplanned readmission identified through medical chart review. The secondary end-point was the composite of multiple cardiovascular events including cardiovascular death and/or unplanned readmission due to cardiovascular disease. We counted the number of these events after the end of the 5-month cardiac rehabilitation. The time period for these events was also calculated as the number of days from the end of the 5-month cardiac rehabilitation to the date of the events.

### 2.5. Cardiac Rehabilitation Program

All patients received comprehensive cardiac rehabilitation during hospitalization and after hospital discharge for five months. Cardiac rehabilitation was initiated when the HF condition was stabilized from the intensive care unit or general wards [12]. The median duration of cardiac rehabilitation initiation from hospitalization for all of studied patients was three days. In the acute phase, we facilitated mobilization and/or ambulation under the monitoring of electrocardiogram (ECG) and vital signs [12]. If patients were able to walk approximately 200 m with independence, they proceeded to inpatient exercise training with the low-moderate intensity [19,20]. Before hospital discharge, cardiologists and medical staff educated patients about self-management including medication, nutrition, and physical activity, and instructed encouragement to participate in outpatient cardiac rehabilitation at least once a week and perform 3–5/week of self-exercise. In outpatient cardiac rehabilitation, exercise training included a 5-min warm-up, 20–40-min aerobic training using a treadmill or bicycle ergometer, and 3-min cool-down periods [19]. Patients also received counselling about lifestyle in ambulatory visits. All exercise training sessions, both inpatient and outpatient, were supervised by trained nurses or physiotherapists, with continuous monitoring assessment.

### 2.6. Statistical Analysis

Clinical variables before and after the 5-month cardiac rehabilitation were compared using the paired Student’s *t*-test or the Wilcoxon signed rank test, as appropriate. We examined the changes in PI_max_ from baseline to five months post-rehabilitation (⊿PI_max_). Patients were divided into two groups based on whether ⊿PI_max_ was positive, and differences in baseline clinical variables were compared between the two groups using the Student’s unpaired *t*-test if parametric, the Mann–Whitney *U*-test if non-normally distributed for continuous variables, and the Chi-square or Fisher’s exact test for categorical variables, as appropriate. The Kruskal–Wallis test and Fisher’s exact test were also used to assess the differences in baseline characteristics and outcomes during the study period based on the time point (years) of study participation. Relationships between ⊿PI_max_ and clinical end-points were analyzed using the Kaplan–Meier method with the log-rank test. To estimate the association between ⊿PI_max_ and a composite of multiple hospitalization and/or death due to all-cause or cardiovascular events, univariate and multivariate Poisson regression models were used. Adjusted incident rate ratios (IRRs) were estimated by analyzing ⊿PI_max_ as a categorical (positive of ⊿PI_max_) or continuous variable (unit increase in 10 cmH_2_O of PI_max_) in separate models. The following clinical confounders at the end of the 5-month cardiac rehabilitation were used as covariates in multivariate analyses: age, sex, BMI, NYHA class, AHEAD score, BNP, the years of study participation, and PI_max_. For missing data on confounders, we performed multiple imputation using the chained equation method, assuming that analyzed data were missing at random. To combine the results from 20 imputed datasets for analysis, Rubin’s formula was used. Time for the Poisson regression models started at the end of the 5-month cardiac rehabilitation. Restricted cubic spline curves with three knots were also used to determine the association between ⊿PI_max_ and clinical events. Subgroup analyses of ⊿PI_max_ in various subgroups relevant to the HF prognosis were performed to assess the potential effect modification on the association of ⊿PI_max_ with clinical events. Trend relationships of clinical events with categories of ⊿PI_max_ per 10 cmH_2_O were examined using the Cochran–Armitage analysis. We also estimated the association between changes in clinical variables following cardiac rehabilitation and all-cause clinical events using multivariate Poisson regression models for each variable. The IRRs were analyzed with meaningful unit change in each variable. To compare the predictive capability of ⊿PI_max_ and the other significant variables in multivariate Poisson analysis for clinical events, the C-index was calculated using multivariate logistic regression models adjusted for confounders used in the Poisson regression model. Continuous variables were reported as the mean ± standard deviation or median with interquartile range, and categorical variables were expressed as patient numbers and their percentages. A two-tailed *P* value of <0.05 was considered significant. All analyses were performed using SPSS 25.0 (IBM, Armonk, NY), Stata version 15.1 (Stata Corp., College Station, TX) and R version 3.1.2 (R Foundation for Statistical Computing, Vienna, Austria).

## 3. Results

### 3.1. Patient Characteristics

The potential study population consisted of 1570 consecutive patients who continued the 5-month cardiac rehabilitation, and those who had received thoracic surgery within the last three months (*n* = 393) or had chronic respiratory diseases (*n* = 140) were excluded from the study. Patients who could not perform the respiratory muscle function test during the observation period (*n* = 581) were also excluded. Consequently, 456 patients with HF were included for analysis in this study.

Overall PI_max_ increased significantly after the 5-month cardiac rehabilitation (Table S1) with positive changes in PI_max_ observed in 326 patients (71.5%). Table 1 shows the baseline patient characteristics in the two groups based on positive changes in PI_max_. The positive change in PI_max_ was significantly associated with lower BNP and PI_max_ and higher prevalence of respiratory muscle weakness at the baseline. However, no statistical differences in other baseline patient characteristics were observed between the two groups. Table S2 shows the differences in baseline characteristics, treatment, and outcomes during the study period based on the time point (years) of study participation. The time point was significantly associated with age, use of diuretics, frequency of outpatient cardiac rehabilitation, and incidence of all-cause events, but not with use of ACE-I/ARB and beta-blockers, change in PI_max_, and incidence of cardiovascular events.

### 3.2. Relationships between Change in Respiratory Muscle Strength and Adverse Clinical Events

**Figure 1:**
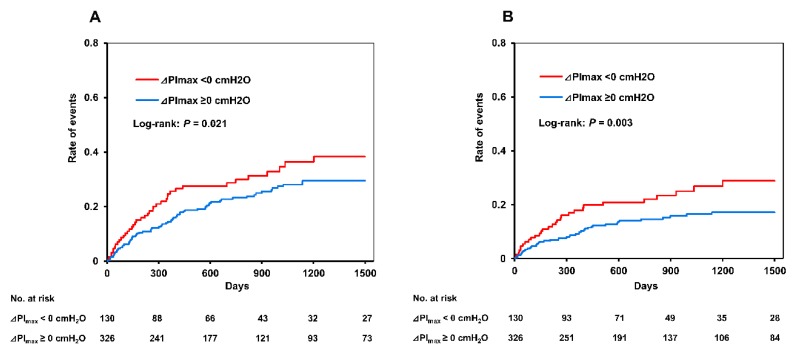
Kaplan–Meier survival curves of the association between change in respiratory muscle strength and clinical events. (**A**) All-cause events and (**B**) cardiovascular events; Red line, patients with ⊿PI_max_ < 0 cmH_2_O; blue line, patients with ⊿PI_max_ ≥ 0 cmH_2_O. PI_max_, maximal inspiratory pressure.

A total of 221 all-cause clinical events and 132 cardiovascular events occurred during the median follow-up period of 1.8 years, and the incidence rate of all-cause events and cardiovascular events was 4.3/100 and 2.4/100 person-years, respectively. Figure 1 shows the Kaplan–Meier survival curves for the two groups. Positive changes in PI_max_ were significantly associated with a lower incidence of all-cause clinical events (log-rank: *p* = 0.021) and cardiovascular events (log-rank: *p* = 0.003).

### 3.3. Poisson Regression Models for Clinical Events

**Figure 2:**
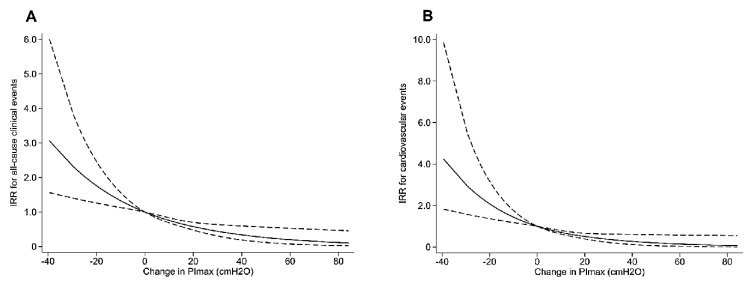
Cubic spline curves of crude relationships between change in respiratory muscle strength and incidence rate of end-points. (**A**) All-cause events and (**B**) cardiovascular events; Dash lines, 95% confidence interval. IRR, incident rate ratio; PI_max_, maximal inspiratory pressure.

Table 2 shows the results of the Poisson regression models of ⊿PI_max_ for all-cause clinical events and cardiovascular events. In the univariate Poisson regression models, ⊿PI_max_ was significantly associated with all-cause clinical events (IRR: 0.75, 95% confidence interval (CI): 0.69–0.82, *p* < 0.001) and cardiovascular events (IRR: 0.71, 95% CI: 0.63–0.79, *p* < 0.001). In the multivariate Poisson regression models adjusted for clinical confounding factors including age, gender, BMI, NYHA class, AHEAD score, BNP, time point of study participation, and PI_max_ values at the end of the 5-month cardiac rehabilitation, ⊿PI_max_ was detected as a significant and independent predictor for all-cause clinical events (adjusted IRR: 0.77, 95% CI: 0.70–0.86, *p* < 0.001) and cardiovascular events (adjusted IRR: 0.72, 95% CI: 0.63–0.82, *p* < 0.001). Positive changes in PI_max_ were also independently associated with decreased all-cause and cardiovascular clinical events (Table 2). Cubic spline analyses clarified linear relationships between changes in PI_max_ and all-cause or cardiovascular events (Figure 2).

**Figure 3:**
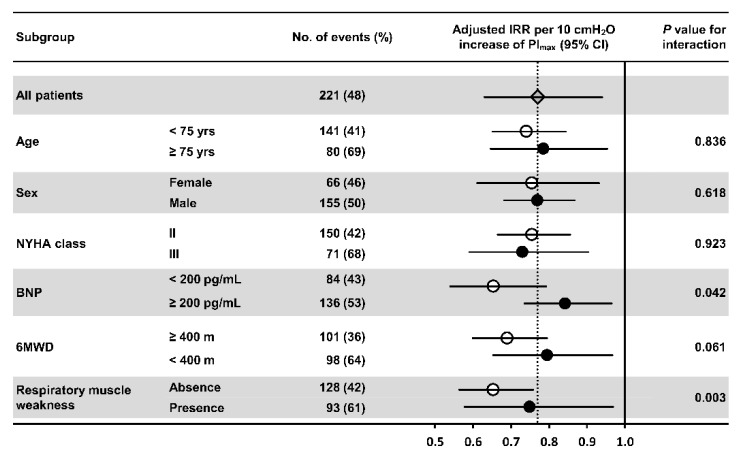
Forest plots of hazard ratios for the association of change in respiratory muscle strength with all-cause clinical events according to major subgroups. Hazard ratios were adjusted for age, sex, BMI, AHEAD score, NYHA class, and BNP at the end of the 5-month cardiac rehabilitation. BMI, body mass index; BNP, brain natriuretic peptide; IRR, incident rate ratio; NYHA, New York Heart Association functional classification; PI_max_, maximal inspiratory pressure; 6MWD, 6-min walk distance.

Figure 3 shows subgroup analyses of ⊿PI_max_ for all-cause clinical events in various subgroups relevant to the HF prognosis. There were no significant interactions in the association of ⊿PI_max_ with the incidence of adverse clinical events across the subgroups of aged >75 years, sex, NYHA class, and6MWD of <400 m at the baseline. Conversely, subgroups with baseline BNP of >200 pg/mL and respiratory muscle weakness showed significant interactions in the association of ⊿PI_max_ with incidence of adverse clinical events. However, higher ⊿PI_max_ was significantly associated with decreased adverse clinical events in all subgroups, even after adjusting for confounding factors used in the multivariate Poisson regression model.

### 3.4. Unadjusted Rates of Clinical Events

**Figure 4:**
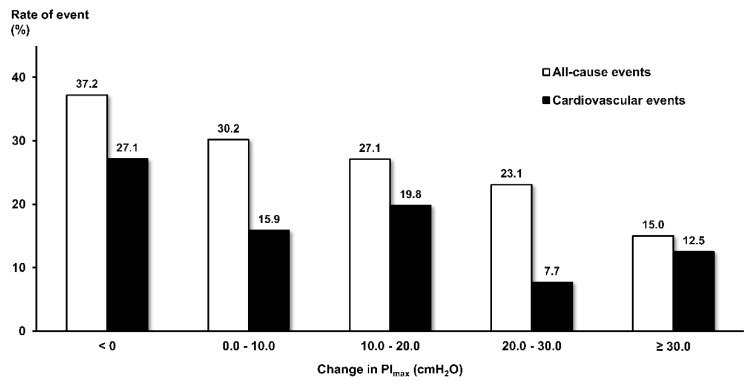
Unadjusted rates of all-cause clinical events and cardiovascular events according to categories of change in PI_max_ per 10 cmH_2_O. White bars, all-cause events; black bars, cardiovascular events. PI_max_, maximal inspiratory pressure.

The unadjusted event rate for ⊿PI_max_ categories per 10 cmH_2_O is shown in Figure 4. The increase in PI_max_ per 10 cmH_2_O had significant trend relationships with a decreased rate of all-cause clinical events (Z = 2.975, *p* = 0.003). There was also a statistically significant trend relationship between the PI_max_ increase and decreased rate of cardiovascular events (Z = 2.906, *p* = 0.004), but the rate showed a fall with increased PI_max_ and subsequent slight rise.

### 3.5. Predictive Significance of Changes in Clinical Variables Following Cardiac Rehabilitation for Clinical Events

**Figure 5:**
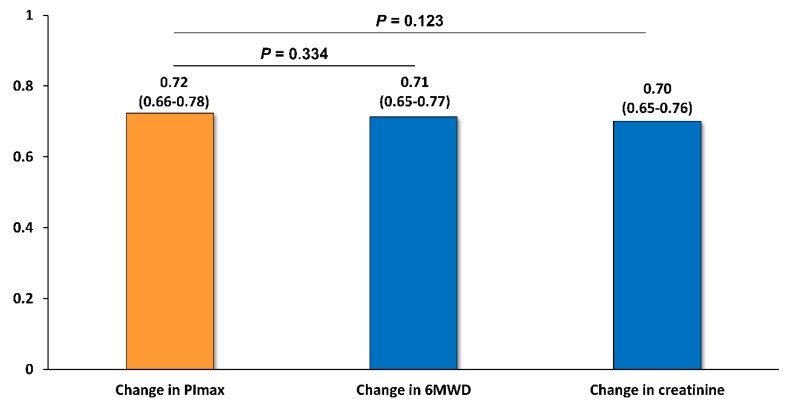
C-index of adjusted models of changes in PI_max_, 6MWD, and creatinine for all-cause clinical events. Data, C-index (95% CI). Models were adjusted for variables used in multivariate Poisson regression analyses. PI_max_, maximal inspiratory pressure; 6MWD, 6-min walk distance.

Table 3 shows the associations between changes in clinical variables and all-cause clinical events. The ⊿6MWD and ⊿creatinine were significantly and independently associated with all-cause clinical events, but the changes in the other variables were not. Figure 5 shows the C-index of the predictive models for clinical events in ⊿PI_max_, ⊿6MWD, and ⊿creatinine. Models were adjusted for age, sex, BMI, AHEAD score, NYHA class, and BNP at the end of 5-month cardiac rehabilitation. The C-index of ⊿PI_max_, ⊿6MWD, and ⊿creatinine were 0.72 (95% CI: 0.66–0.78), 0.71 (95% CI: 0.65–0.77), and 0.70 (0.65-0.76), respectively, and there were no statistical differences between the three predictive models.

## 4. Discussion

The novel findings in the present study are as follows. First, changes in respiratory muscle strength following the cardiac rehabilitation significantly and independently predicted the incidence of adverse clinical events in patients with HF. Second, positive changes in PI_max_ of 10 cmH_2_O following the cardiac rehabilitation were associated with 23% decrease of adverse clinical events.

To the best of our knowledge, this study is the first to demonstrate that the longitudinal change of respiratory muscle strength is a significant indicator of prognosis in patients with HF. Our previous study reported on the respiratory muscle strength as a significant predictor for prognosis in patients with HF with both reduced ejection fraction (HFrEF) and preserved ejection fraction (HFpEF) [7]. In general, decreased respiratory muscle strength is associated with reduced pulmonary function [6,21], a known risk factor for cardiovascular event including HF [22]. Conversely, Habedank and colleagues showed that PI_max_, generally measured as a respiratory muscle function, was not an independent predictor of prognosis because it varied according to gender, BMI, and cachexia in patients with severe HFrEF [11]. A recent study has shown impaired respiratory muscle oxygenation during exercise in patients with HF, leading to breathlessness and decreased quality of life [23]. In the present study, the higher change in PI_max_ was associated with lower respiratory muscle strength at the baseline. In addition, positive changes in PI_max_ showed a trend relationship with declined clinical events, even after adjusting for gender, BMI, comorbidities, and HF severity. These relationships remained significant even in subgroups including elderly patients, those with lower functional capacity, or respiratory muscle weakness at baseline. These results suggest that the respiratory muscle weakness is likely to be improved by cardiac rehabilitation, and the repeated measurement of respiratory muscle strength might be more important than a single measurement to assess the clinical condition in patients with HF.

Several studies have indicated that increased respiratory muscle strength contributes to the improvement of respiratory muscle fatiguability, exercise tolerance, and quality of life [9,24]. Chiappa et al. reported the effects of inspiratory muscle training on peripheral blood flow during respiratory muscle fatigue stress in patients with HF [25]. They demonstrated that inspiratory muscle training improved peripheral muscle blood flow with decreased peripheral vascular resistance during respiratory muscle fatigue. In general, respiratory muscle fatigue induces sympathetic vasomotor outflow, resulting in the increased peripheral vascular resistance [26]. Conversely, increased inspiratory muscle strength augments tidal volume and consequently improves input to the pulmonary stretch receptor and autonomic nervous activity [27]. This improvement is documented in combination of reduced sympathetic activity and elevated parasympathetic activity, and thereby attenuates vascular resistance and increases peripheral blood flow [27]. These results are potential mechanisms of inspiratory muscle training to improve exercise tolerance and are likely correlated with our finding that increased respiratory muscle strength may improve the prognosis of patients with HF.

This study provides clinical implications that change in respiratory muscle strength is identified as a significant clinical marker for patients with HF. Previous studies have documented that the trajectory of 6MWD or renal function are associated with morbidity and mortality in these patients [28,29]. We revealed that the predictive capability of changes in respiratory muscle strength following cardiac rehabilitation was relatively higher or comparable to that of changes in 6MWD or renal function. In general, measurement of respiratory muscle strength is easy to perform in clinical practice. Therefore, respiratory muscle strength changes might be a useful marker to assess the effects of HF treatment. Furthermore, the respiratory muscle strength can be modified with exercise training including inspiratory muscle training in patients with HF [9,30]. Our results suggest the potential benefits of an increased respiratory muscle strength on the prognosis in patients with HF.

However, some limitations remain to be considered in the present study. First, as this was a single-center study that only included Japanese patients and the sample size was relatively small, whether these results can be applied to patients with HF in other hospitals or other populations remains to be elucidated. In addition, external validity could have been reduced, given that half of the potential study population was excluded from the analysis. Multivariate analyses were also performed using multiple confounders, which might increase the false-positive rates (type I error). Therefore, future multicenter studies are required to reveal the validity and reliability of change in respiratory muscle strength as predictors of prognosis in patients with HF. Second, this was not a randomized control trial. Hence, whether exercise training per se increased the respiratory muscle strength and decreased clinical events remains to be investigated. Further interventional study is required to investigate whether increased respiratory muscle strength due to exercise training improves the prognosis of patients with HF. Third, the period of data review was relatively long-term, 8.5 years, and the time point of study participation was associated with age, use of diuretics, and frequency of outpatient CR. Nevertheless, in the multivariate regression analyses, impact of the time point of study participation on relationships between change in respiratory muscle strength and outcomes was not observed. Fourth, in this study, while the respiratory muscle weakness was defined with 70% predicted value of PI_max_, there was limited evidence using the % PI_max_ as a cut-off value for the weakness. As the more robust lower limit of normal values of PI_max_ has been suggested by the ATS/ERS statement in other patient groups [16], we consider it important to clarify the meaningful cut-off value of respiratory muscle weakness for patients with HF in the future.

## 5. Conclusions

Change in respiratory muscle strength following cardiac rehabilitation significantly and independently predicted clinical events in patients with HF. The 10 cmH_2_O increase of PI_max_ was significantly associated with a 23% decreased incidence of clinical events.

## Figures and Tables

**Table 1:** Baseline patient characteristics in the two groups based on change in PI_max._

Groups	Overall	⊿PI_max_ ≤ 0 cmH_2_O	⊿PI_max_ > 0 cmH_2_O	*p* Value
***n***	456	130	326	
Age, y.o.	68 (57–75)	69 (59–74)	67 (55–75)	0.285
Gender, *n* (%)				
Female	144 (31.6)	38 (29.2)	106 (32.5)	0.577
Male	312 (68.4)	92 (70.8)	220 (67.5)	
BMI, kg/m^2^	23.0 ± 4.1	22.8 ± 3.7	23.1 ± 4.3	0.478
HR, beats/min	82 ± 23	84 ± 25	81 ± 22	0.224
sBP, mm Hg	123 ± 29	123 ± 32	123 ± 28	0.839
dBP, mm Hg	72 ± 19	72 ± 21	71 ± 19	0.638
Medical History, *n* (%)				
Ischemic Heart Disease	240 (52.6)	72 (55.4)	168 (51.5)	0.469
Cardiomyopathy	95 (20.8)	25 (19.2)	70 (21.5)	0.702
Atrial Fibrillation	79 (17.3)	29 (22.3)	50 (15.3)	0.099
Hypertension	298 (65.4)	89 (68.5)	209 (64.1)	0.446
Dyslipidemia	292 (64.0)	88 (67.7)	204 (62.6)	0.332
Diabetes Mellitus	174 (38.2)	49 (37.7)	125 (38.3)	0.915
Chronic Kidney Disease	261 (57.4)	79 (60.8)	182 (56.0)	0.401
Prior Admission for HF, *n* (%)	101 (22.1)	34 (26.2)	67 (20.6)	0.212
NYHA Class, *n* (%)				
II	353 (77.8)	103 (80.5)	250 (76.7)	0.452
III	101 (22.2)	25 (19.5)	76 (23.3)	
LVEF, %	46.3 ± 15.2	46.4 ± 14.7	46.3 ± 15.4	0.967
LVEF groups, *n* (%)				
<40%	148 (33.2)	45 (35.2)	103 (32.4)	0.806
40–50%	189 (42.4)	54 (42.2)	135 (42.5)	
>50%	109 (24.4)	29 (22.7)	80 (25.2)	
Smoking History, *n* (%)	259 (56.8)	77 (59.2)	182 (55.8)	0.531
Pack-Years	9.0 (0.0–35.0)	10.0 (0.0–40.0)	8.3 (0.0–33.0)	0.401
Medications, *n* (%)				
ACE-I or ARB	392 (86.0)	116 (89.2)	276 (84.7)	0.234
Beta-Blockers	374 (82.0)	108 (83.1)	266 (81.6)	0.788
Diuretic	308 (67.5)	92 (70.8)	216 (66.3)	0.377
Hemoglobin, g/dL	12.8 ± 2.3	13.0 ± 2.5	12.7 ± 2.2	0.168
Albumin, g/dL	3.7 ± 0.5	3.7 ± 0.5	3.7 ± 0.5	0.837
Creatinine, g/dL	0.98 (0.80–1.21)	0.99 (0.84–1.23)	0.96 (0.80–1.21)	0.395
eGFR, mL/min/1.73m^2^	55.7 ± 21.5	55.1 ± 22.2	56.0 ± 21.2	0.685
BNP, pg/mL	227.7 (113.0–435.4)	269.7 (129.7–528.5)	213.2 (106.7–385.8)	0.030
AHEAD Score	1.6 ± 1.2	1.6 ± 1.2	1.6 ± 1.2	0.659
Six-min Walk Distance, m	446 (360–510)	435 (355–525)	448 (363–507)	0.852
%FVC, %	80.0 ± 18.1	80.6 ± 19.4	79.8 ± 17.5	0.661
FEV_1_/FVC, %	77.5 ± 9.5	76.8 ± 8.0	77.8 ± 10.0	0.349
PI_max_, cmH_2_O	58.5 ± 27.1	67.5 ± 27.0	54.9 ± 26.4	<0.001
Respiratory Muscle Weakness, *n* (%)	148 (33.0)	26 (20.3)	122 (38.1)	<0.001

Values are mean ± SD, or median (interquartile range). ACE-I, angiotensin convertor enzyme inhibitor; ARB, angiotensin II receptor blocker; BMI, body mass index; BNP, brain natriuretic peptide; dBP, diastolic blood pressure; eGFR, estimated glomerular filtration rate; FEV_1_, forced expiratory volume in 1-s; FVC, forced vital capacity; HF, heart failure; HR, heart rate; LVEF, left ventricular ejection fraction; NYHA, New York Heart Association; PI_max_, maximal inspiratory pressure; sBP, systolic blood pressure.

**Table 2:** Poisson regression models of changes in PI_max_ for adverse clinical events.

		Univariate Analysis			Multivariate Analysis		
		IRR	95% CI	*p* Value	IRR	95% CI	*p* Value
**All-Cause Events**							
⊿PI_max_ increase of 10 cmH_2_O		0.75	0.69–0.82	<0.001	0.77	0.70–0.86	<0.001
⊿PI_max_	≤0 cmH_2_O	1.00	Reference		1.00	Reference	
	>0 cmH_2_O	0.52	0.40–0.68	<0.001	0.70	0.52–0.93	0.014
**Cardiovascular Events**							
⊿PI_max_ increase of 10 cmH_2_O		0.71	0.63–0.79	<0.001	0.72	0.63–0.82	<0.001
⊿PI_max_	≤0 cmH_2_O	1.00	Reference		1.00	Reference	
	>0 cmH_2_O	0.39	0.28–0.55	<0.001	0.52	0.36–0.75	<0.001

Multivariate analyses were adjusted for age, gender, BMI, time point of study participation, AHEAD score, NYHA class, BNP, and PI_max_. IRR, incident rate ratio; CI, confidence interval; PI_max_, maximal inspiratory pressure.

**Table 3:** Poisson regression models of changes in clinical variables for all-cause events.

	Adjusted IRR	Unit Changes	95% CI	*p* Value	
⊿BMI	1.03	1 kg/m^2^	0.95–1.11		0.514
⊿HR	1.00	5 beats/min	0.99–1.01		0.826
⊿sBP	1.00	5 mm Hg	0.99–1.04		0.856
⊿dBP	1.04	5 mm Hg	0.99–1.10		0.125
⊿Hemoglobin	0.96	1 g/dL	0.89–1.05		0.379
⊿Albumin	0.98	0.1 g/dL	0.95–1.01		0.265
⊿Creatinine	0.90	0.1 g/dL	0.89–0.91		<0.001
⊿eGFR	1.00	1 mL/min/1.73m^2^	0.99–1.01		0.825
⊿BNP	1.00	10 pg/mL	1.00–1.01		0.096
⊿6MWD	0.93	10 m	0.91–0.95		0.019
⊿%FVC	0.98	5%	0.92–1.05		0.595
⊿FEV1/FVC	1.04	5%	0.95–1.14		0.344

IRRs were adjusted for age, gender BMI, time point of study participation, AHEAD score, NYHA class, and BNP. BMI, body mass index; BNP, brain natriuretic peptide; BP, blood pressure; CI, confidence interval; eGFR, estimated glomerular filtration rate; FEV_1_, forced expiratory volume in 1-s; FVC, forced vital capacity; IRR, incident rate ratio; HR, heart rate; 6MWD, 6-min walk distance.

## Supplementary Materials

The following are available online at https://www.mdpi.com/2077-0383/9/4/952/s1, Table S1: Changes in patient characteristics before and after the 5-month cardiac rehabilitation; Table S2: Differences in baseline characteristics, treatment, and outcomes during the study period based on the time point of study participation.
