# Supplementary material for: Changes in Respiratory Muscle Strength Following Cardiac Rehabilitation for Prognosis in Patients with Heart Failure
Source: J Clin Med. 2020 Mar 30;9(4):952. doi: 10.3390/jcm9040952 (PMC7230659; doi:10.3390/jcm9040952)

Changes in respiratory muscle strength following cardiac rehabilitation predict clinical events

Setting

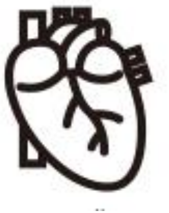

Heart failure

456 patients

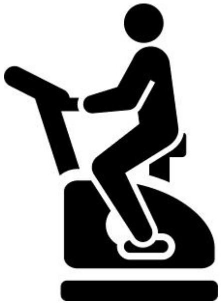

5-month

Cardiac rehabilitation

Findings

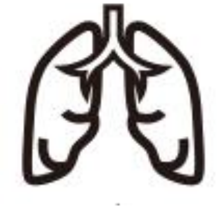

- **Maximal inspiratory pressure (PI_{max})**

  8.1 cmH_2O (median) ↑

- **Positive changes in PI_{max} per 10 cmH_2O**

  All-cause clinical events: 23% ↓*

  Cardiovascular events: 28% ↓*

  *Adjusted for clinical confounders

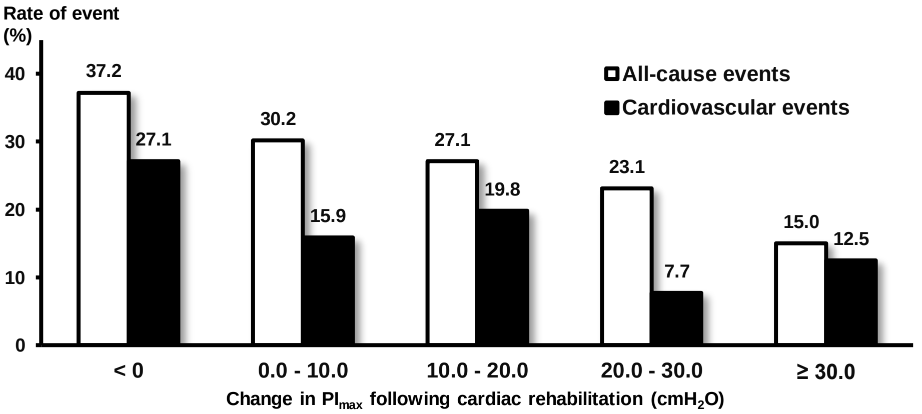

Supplement: Supplementary file 1 [file jcm-09-00952-s001.pdf]
